# Treatment patterns and burden of uncomplicated urinary tract infection in England: a retrospective cohort study

**DOI:** 10.3399/BJGPO.2024.0214

**Published:** 2025-04-24

**Authors:** Mark H Wilcox, Dave Heaton, Aruni Mulgirigama, Ashish V Joshi, Viktor Chirikov, Daniel C Gibbons, David Webb, Xiaocong L Marston, Myriam NA Alexander, Fanny S Mitrani-Gold

**Affiliations:** 1 Department of Microbiology, University of Leeds, Leeds, UK; 2 Leeds Teaching Hospitals NHS Trust, Leeds, UK; 3 OPEN Health, Marlow, UK; 4 GSK, London, UK; 5 GSK, Collegeville, PA, US; 6 OPEN Health, Bethesda, MD, US

**Keywords:** general practice, community-acquired infections, urinary tract infections, cystitis, lower urinary tract symptoms

## Abstract

**Background:**

Uncomplicated urinary tract infections (uUTIs) are common bacterial infections.

**Aim:**

To evaluate the burden of uUTI in England for 1) potential determinants of disease progression; 2) extent and impact of antimicrobial prescribing non-concordant with treatment guidelines; and 3) healthcare burden and economic costs.

**Design & setting:**

Retrospective cohort study utilising patient data (January 2017–February 2020) from the Clinical Practice Research Datalink (CPRD) linked to English Hospital Episode Statistics.

**Method:**

Female patients aged ≥12 years with a new uUTI between 2018 and 2019, ≥14 months’ continuous CPRD enrolment (≥12 months baseline, ≥2 months follow-up), and ≥1 oral antibiotic prescription ±5 days of uUTI diagnosis were included. Baseline characteristics were described in patients with or without disease progression (hospitalisation for acute pyelonephritis, bacteraemia, or sepsis). Treatment non-concordance with current English guidelines was assessed. Burden (all-cause and urinary tract infection-related healthcare resource use [HCRU] and costs) was evaluated in a 1:1 age and comorbidity-matched uUTI-free cohort.

**Results:**

Of 120 519 patients, 207 (0.2%) had disease progression requiring hospitalisation (during index uUTI episode); determinants included older age, index uUTI home consultation, prior hospitalisation, and medications prescribed for comorbid conditions in the prior 12 months (*British National Formulary* classes: cardiovascular system, eye, and other drugs and preparations). Non-concordant treatment was observed in 43.5% of patients. All-cause HCRU burden and costs were significantly higher in patients with uUTI versus age and comorbidity-matched controls (*P*<0.001) at 28 days (£160.06 versus £37.63) and in the 12-month follow-up (£1206.77 versus £460.97).

**Conclusion:**

All-cause HCRU burden and costs were significantly higher in patients with uUTI versus matched controls (*P*<0.001). Hospitalisation for acute pyelonephritis, bacteraemia, or sepsis following uUTI was uncommon.

## How this fits in

Our study provides data on the burden of uncomplicated urinary tract infection (uUTI) in England, where there is currently a paucity of data on determinants of disease progression, the prevalence and impact of prescribing that is non-concordant with prescribing guidelines, and economic burden. Our findings indicate that disease progression is not common among patients with uUTI. Higher all-cause healthcare resource use (HCRU) and higher costs are common among patients with uUTI compared with age- and comorbidity-matched controls. Increased awareness of the healthcare burden of uUTI in England may be informative for future optimisation of care.

## Introduction

uUTI is a community-acquired bacterial infection, predominantly caused by *Escherichia coli* (*E. coli*).^1^ uUTIs are characterised by dysuria, lower abdominal pain, urinary frequency, and urinary urgency in patients without urological abnormalities or complicating factors.^2,3^ Rarely, uUTIs can progress to more serious infections such as acute pyelonephritis, bacteraemia, or sepsis.^4^ Limited evidence exists on correlating factors associated with uUTI disease progression.^5–7^

Prescribing in England follows National Institute for Health and Care Excellence (NICE) urinary tract infection (UTI) guidelines (last published in 2018).^8^ However, despite financial incentives, many healthcare providers in England still prescribe non-concordant treatments for patients with uUTI,^9^ which can increase risk of treatment failure, disease progression, and antimicrobial resistance, causing increased HCRU and costs.^10,11^

This study of community-acquired uUTI among female patients in England had three objectives: 1) to identify determinants of disease progression requiring hospitalisation for sepsis, acute bactaraemia, or pyelonephritis; 2) to characterise treatment patterns and extent of guideline non-concordant antimicrobial treatment and its effect on risk of disease progression; and 3) to evaluate the healthcare burden and economic costs of uUTI in England.

## Method

### Study design

This retrospective cohort study used patient data from an overall study period of 1 January 2017–29 February 2020. Data were selected from the Clinical Practice Research Datalink (CPRD) database, which contains coded and anonymised electronic health record data from primary care practices in England,^12^ and Hospital Episode Statistics (HES), which contains details of admissions and emergency attendances at NHS hospitals in England.

Patients were female, aged ≥12 years, and with a new diagnosis of community-acquired uUTI, defined as the earliest observed uUTI code in primary care without an adjacent uUTI code ≤28 days prior to index (codes provided in Supplementary Table S1). Study design and analysis periods are in Supplementary Figure S1. uUTI episodes occurring between 1 January 2018 and 31 December 2019 (identification period) were included. Patients had ≥1 oral antibiotic prescription ±5 days of index and ≥14 months’ continuous enrolment in the CPRD (≥12 months pre-index to capture baseline characteristics and ≥2 months post-index for outcome assessment) with data linked to HES. Patients were excluded if they had medical Read codes denoting structural or functional urological abnormalities, urological procedures, complicating comorbidities during baseline (for example, pregnancy, complicated or uncontrolled diabetes, and immunosuppression), hospitalisation or accident and emergency (A&E) department visits ≤28 days pre-index, or intravenous antibiotic use as index uUTI therapy (see Supplementary Table S2).

To analyse economic burden, a subgroup of patients with uUTIs with ≥12 months post-index enrolment were included. Controls were females aged ≥12 years, with a primary care visit in the CPRD between 1 January 2018 and 31 January 2019 (identification period), and no UTI diagnosis during baseline and follow-up periods (see Supplementary Figure S1).

### Variables

For objective 1 (determinants of disease progression requiring hospitalisation for sepsis, acute bacteraemia, or pyelonephritis) patient-level (demographic and clinical), region-level (NHS regions), and practice-level variables in the 12-month baseline period were characterised descriptively in patients with or without disease progression. To provide information on comorbidities, receipt of medication(s) in the 12 months prior to index was described by *British National Formulary* (*BNF*) drug classes.

To characterise the extent of non-concordant uUTI prescribing and its effect on risk of disease progression (objective 2), patient exposures to antimicrobial therapies during the 28-day index episode were identified in the CPRD and compared with NICE lower UTI antimicrobial prescribing guidelines (NG109) (see Supplementary Table S3). Patients were categorised as ‘concordant’ or ‘non-concordant’ with index antibiotic treatment based on prescribing information. Concordant or non-concordant treatment criteria are in Supplementary Information S1. Patients were further stratified by represcription status (‘yes’ or ‘no’), within 28 days of index. Patients receiving a re-prescription (any antibiotic prescription [same antibiotic or switch] between days 4 and 28 post-index) had the index episode extended by 28 days from re-prescription date, to capture 30-day health outcomes in all patients including those switching prescriptions.

To evaluate the uUTI healthcare and economic burden in England (objective 3), using a patient subset with ≥12 months follow-up post-index, HCRU and costs were compared versus a reference (control) patient population without UTI. Patients were matched 1:1 on age and baseline Charlson Comorbidity Index (CCI) score (0, 1, 2, 3, and ≥4). All-cause HCRU and costs were evaluated for the 28-day index uUTI episode and 12 months post-index; these included primary care consultations, specialist consultations, A&E attendance, hospital admissions, and high dependency unit (HDU) or intensive therapy unit (ITU) admissions. Costs were estimated for primary care (*BNF* drug costs and Personal Social Services Research Unit reference costs) and secondary care (Healthcare Resource Groups linked to Payment by Results tariff).^13–16^

Supplementary Table S4 provides details of all measured variables.

### Statistical analysis

For objectives 1 and 2, demographics, clinical characteristics, and antibiotic treatment during baseline were summarised descriptively.

For objective 1, determinants of hospitalisation (owing to acute pyelonephritis, bacteraemia, or sepsis) were assessed via univariate and multivariate logistic regression models (see Supplementary Information S1) using Hosmer and Lemeshow’s purposeful selection of variables.^17,18^ Unmeasured confounding was assessed via e-values for odds ratios (ORs), representing the minimum strength of association an unmeasured confounder would require with both treatment and outcome, conditional on the measured covariates.

For objective 2*,* effect of receiving guideline non-concordant treatment on risk of hospitalisation was determined using an inverse probability of treatment weighted multivariate logistic regression model. To mitigate non-random allocation of treatment, inverse probability of treatment weights were created using propensity score methodology (covariate balancing propensity score) to balance covariates (age at index, diagnosis year, index uUTI as a home visit, history of recurrent UTI, practice size, region, mild or moderate renal impairment, and Index of Multiple Deprivation [IMD] quintile) among patients receiving concordant versus non-concordant treatment.

For objective 3, the association between uUTI and HCRU and costs was assessed using zero-inflated Poisson and gamma regression models. This was required because most participants did not require hospital attendance, therefore had no HCRU and costs, leading to data overdispersion.

Details on how costs were generated, sample size, and power calculations for all objectives are in Supplementary Information S1.

## Results

### Baseline characteristics

Overall, the study included 120 519 female patients with uUTI in England (Table 1); de-identified patient data were obtained from the CPRD Aurum and GOLD databases linked to HES.^12,19^ Mean age was 52.1 (standard deviation [SD] 21.6) years. More patients lived in urban (86.8%) versus rural areas (13.2%) and were seen at large practices (51.6%), with the largest proportion living in North West England (20.9%).

**Table 1.** Patient-, region-, and practice-level demographic and clinical characteristics of female patients with uUTI overall, and patients with uUTI with and without hospitalisation for disease progression in England

Characteristic	Statistic or category	All patients (*N* = 120 519)	Hospitalised during post-index uUTI follow-up period (*n* = 207)	No hospitalisation (*n* = 116 586)
**Patient level**				
Age at diagnosis, years	Mean (SD)	52.1 (21.6)	67.3 (20.2)	52.9 (21.1)
	Median (Q1; Q3)	52.0 (33.0; 71.0)	74.0 (55.0; 83.0)	53.0 (35.0; 71.0)
Pooled age at diagnosis, *n* (%)	<50 years (pre-menopause analogue)	55 789 (46.3)	39 (19.0)	52 438 (45.0)
	≥50 years (post-menopause analogue)	64 730 (53.7)	168 (81.0)	64 148 (55.0)
Year of diagnosis, *n* (%)	2018	64 964 (53.9)	126 (61.0)	62 827 (54.0)
	2019	55 555 (46.1)	81 (39.0)	53 759 (46.0)
Race or ethnicity, *n* (%)	Asian or Asian British	5334 (4.4)	6 (2.9)	5161 (4.4)
	Black or Black British	3444 (2.9)	6 (2.9)	3287 (2.8)
	Chinese or other group^a^	2072 (1.7)	<5	1995 (1.7)
	Ethnic group not recorded	24 566 (20.4)	43 (21.0)	22 781 (20.0)
	Mixed^b^	42 865 (35.6)	77 (37.0)	42 022 (36.0)
	White	42 238 (35.0)	73 (35.0)	41 340 (35.0)
Index uUTI consultation as a home visit, *n* (%)	Yes	1838 (1.5)	22 (11.0)	1785 (1.5)
Hospital or A&E admissions in prior year	Mean (SD)	0.3 (0.8)	0.8 (1.5)	0.3 (0.8)
Number of A&E attendances in prior year	Mean (SD)	0.5 (1.3)	0.8 (1.4)	0.5 (1.3)
History of recurrent uUTI, *n* (%)	Yes	5054 (4.2)	11 (5.3)	4937 (4.2)
Any prior exposure to antimicrobial treatment	Yes	NA	150 (72.0)	70 274 (60.0)
Number of antibiotic prescriptions	Mean (SD)	1.05 (1.2)	1.4 (1.3)	1.1 (1.2)
Smoking status	Non-smoker	72 944 (62.2)	119 (57.0)	72 546 (62.0)
	Former smoker	27 218 (23.2)	62 (30.0)	27 084 (23.0)
	Smoker	17 046 (14.5)	26 (13.0)	16 956 (15.0)
Menopausal, *n* (%)	Yes	34 276 (28.4)	60 (29.0)	34 072 (29.0)
Obese, *n* (%)	Yes	13 975 (11.6)	33 (16.0)	13 858 (12.0)
CCI	Mean (SD)	0.2 (0.5)	0.4 (0.7)	0.2 (0.5)
CCI category, *n* (%)	0	99 665 (82.7)	134 (65.0)	95 944 (82.0)
	1	18 199 (15.1)	58 (28.0)	18 007 (15.0)
	≥2	2653 (2.2)	12 (5.8)	2635 (2.3)
Mild or moderate renal impairment, *n* (%)	Yes	2282 (1.9)	10 (4.8)	2262 (1.9)
Liver disease, *n* (%)	Yes	98 (<1)	<5	97 (<1)
Controlled diabetes with HbA1c levels <6.5/7%, *n* (%)	Yes	2473 (2.1)	14 (6.8)	2451 (2.1)
Medications for comorbid conditions in the prior 12 months by *BNF* class, *n* (%)	1: Gastrointestinal system	47 822 (39.7)	122 (59.0)	47 129 (40.0)
	2: Cardiovascular system	45 101 (37.4)	138 (67.0)	44 561 (38.0)
	3: Respiratory system	28 342 (23.5)	66 (32.0)	27 841 (24.0)
	4: Central nervous system	60 403 (50.1)	149 (72.0)	59 459 (51.0)
	5: Infections	74 402 (61.7)	150 (72.0)	72 419 (62.0)
	6: Endocrine system	31 906 (26.5)	86 (42.0)	31 445 (27.0)
	7: Obstetrics, gynaecology, and urinary tract disorders	34 540 (28.7)	36 (17.0)	33 703 (29.0)
	8: Malignant disease and immunosuppression	1654 (1.4)	7 (3.4)	1638 (1.4)
	9: Nutrition and blood	29 307 (24.3)	87 (42.0)	28 763 (25.0)
	10: Musculoskeletal and joint diseases	28 542 (23.7)	73 (35.0)	28 061 (24.0)
	11: Eye	12 888 (10.7)	49 (24.0)	12 642 (11.0)
	12: Ear, nose, and oropharynx	17 223 (14.3)	25 (12.0)	16 838 (14.0)
	13: Skin	32 808 (27.2)	73 (35.0)	31 765 (27.0)
	14: Immunological products and vaccines	13 713 (11.4)	38 (18.0)	13 548 (12.0)
	15: Anaesthesia	3630 (3.0)	6 (2.9)	3498 (3.0)
	18: Preparations used in diagnosis	0 (0.0)	0 (0.0)	0 (0.0)
	19: Other drugs and preparations^c^	508 (<1)	5 (2.4)	488 (<1)
	NA: Prescription could not be mapped to *BNF*	3464 (2.9)	—	—
**Region level**				
Quintiles of Index of Multiple Deprivation, *n* (%)	Q1 (least deprived)	27 749 (23.0)	42 (20.0)	26 869 (23.0)
	Q2	24 277 (20.1)	39 (19.0)	23 597 (20.0)
	Q3	22 992 (19.1)	38 (18.0)	22 273 (19.0)
	Q4	22 765 (18.9)	46 (22.0)	22 085 (19.0)
	Q5 (most deprived)	22 658 (18.8)	42 (20.0)	21 762 (19.0)
	Missing	78 (<1)	—	—
Rural–urban area classification, *n* (%)	Rural	15 883 (13.2)	30 (14.0)	15 416 (13.0)
	Urban	104 636 (86.8)	177 (86.0)	101 170 (87.0)
Region, *n* (%)	East Midlands	2504 (2.1)	<5	2440 (2.1)
	East of England	4816 (4.0)	8 (3.9)	4664 (4.0)
	London	19 500 (16.2)	28 (14.0)	18 898 (16.0)
	North East	2813 (2.3)	9 (4.3)	2739 (2.3)
	North West	25 150 (20.9)	54 (26.0)	24 269 (21.0)
	South Central	17 320 (14.4)	32 (15.0)	16 826 (14.0)
	South East Coast	11 152 (9.3)	12 (5.8)	10 788 (9.3)
	South West	12 960 (10.8)	17 (8.2)	12 490 (11.0)
	West Midlands	20 625 (17.1)	36 (17.0)	19 884 (17.0)
	Yorkshire and the Humber	3675 (3.0)	7 (3.4)	3588 (3.1)
**Practice level**				
Practice size, *n* (%)	Small (<8000 patients)	31 762 (26.4)	56 (27.0)	30 783 (26.0)
	Medium (8000–11 000 patients)	26 189 (21.7)	44 (21.0)	25 377 (22.0)
	Large (>11 000 patients)	62 222 (51.6)	107 (52.0)	60 426 (52.0)
	Missing	346 (<1)	—	—

Patients with missing data were excluded. Percentage data for each row have been calculated using independent *n*-values (data not able to be shown, cells with *n*<5 suppressed per CPRD data reporting requirements), hence the slight discrepancy in some percentage calculations versus their associated, rounded, *n*-values. ^a^The ‘Other’ category includes patients of Asian or Asian British, Black or Black British, Chinese, or other race or ethnicity. ^b^The ‘Mixed’ category refers to mixed European ethnicity. ^c^Other drugs and preparations include: alcohol, wines, and spirits; selective preparations; single substances; other preparations; acids; base, diluent, suspending agents, and stabilisers; colouring, flavouring, and sweetening agents; disinfectants, preservatives, and sterilising agents; cordials and soft drinks; waters; and other gases. A&E = accident and emergency. *BNF* = *British National Formulary*. CCI = Charlson Comorbidity Index. CPRD = Clinical Practice Research Datalink. HbA1c = glycated haemoglobin. IQR = interquartile range. NA = not applicable. Q = quartile or quintile (as specified). SD = standard deviation. uUTI = uncomplicated urinary tract infection.

### Objective 1: Risk of hospitalisation

Overall, 207 of 120 519 patients (0.2%) were hospitalised during their index uUTI episode (Table 1). The most common cause of hospitalisation was sepsis (*n* = 183), followed by acute pyelonephritis (*n* = 29); five patients were hospitalised with both sepsis and acute pyelonephritis, and none were hospitalised with bacteraemia (data not shown).

Compared with the non-hospitalised group, patients in the hospitalised group were older (mean age 67.3 [SD 20.2] versus 52.9 [SD 21.1] years), more frequently had prior antimicrobials (72.0% versus 60.0%), had a home visit for the index consultation (11% versus 2%), and higher overall comorbidity burden (Table 1).

After excluding patients with missing data, univariate and multivariate analyses included 116 793 patients, of whom 207 (0.2%) were hospitalised. In the univariate analysis, patient-level variables identified as significant determinants of hospitalisation were: age, index uUTI home consultation, number of hospital admissions in prior year, number of A&E attendances in prior year, prior antibiotic treatment, cumulative antibiotic prescriptions, non-smoking status, CCI score, mild or moderate renal impairment, controlled diabetes, and prior medications of certain *BNF* classes; all *P*<0.05 (see Supplementary Table S5).

After adjusting for covariables, older age, index uUTI as a home consultation, prior hospital admission, and receipt of medication(s) for comorbidities in the 12 months prior to index (*BNF* classes: cardiovascular system, eye, and other drugs and preparations) were identified as determinants of hospitalisation (owing to uUTI progression) (Table 2). Patients with history of a prescription to *BNF* class 7 (‘obstetrics, gynaecology, and urinary tract disorder’) had 35% lower odds of hospitalisation versus those not prescribed this medication class.

**Table 2.** Determinants of hospitalisation owing to disease progression (objective 2)

Variable description^a^	Statistic or category	Multivariate OR	95% **CI**	*P*-value	e-value
**Age at diagnosis, years**	Increasing age per year	1.02	1.01 to 1.03	<0.001	1.16
**Index uUTI consultation as a home visit**	Yes	3.25	1.98 to 5.12	<0.001	5.96
**Hospital stays (from day 29 to day 365 prior to index uUTI)**	Number of admissions in prior year	1.22	1.12 to 1.31	<0.001	1.73
**Medications for comorbid conditions in the prior 12 months by *BNF* class**	Cardiovascular system	1.66	1.17 to 2.35	<0.01	2.43
	Obstetrics, gynaecology, and urinary tract disorders	0.65	0.45 to 0.93	<0.05	2.41
	Eye	1.52	1.08 to 2.11	<0.05	5.49
	Other drugs and preparations^a^	3.02	1.06 to 6.74	<0.05	2.70

^a^Other drugs and preparations include: alcohol, wines, and spirits; selective preparations; single substances; other preparations; acids; base, diluent, suspending agents, and stabilisers; colouring, flavouring, and sweetening agents; disinfectants, preservatives, and sterilising agents; cordials and soft drinks; waters; and other gases. *BNF = British National Formulary*. OR = odds ratio. uUTI = uncomplicated urinary tract infection.

### Objective 2: Non-concordant treatment

Overall, 52 460 (43.5%) patients received first-line treatment not concordant with NICE recommendations (see Supplementary Table S6).^8^ Nitrofurantoin and trimethoprim were the most common therapies prescribed across both adult and adolescent patients as first, second, and third-line therapy (Table 3).

**Table 3.** Antimicrobials prescribed by age and line of therapy among non-pregnant females with uUTI in England (*n* = 120 519)

**Single-agent therapy**	**Metric**	**First-line therapy, *n* = 118 540 (100%)**	**Second-line therapy, *n* = 31 425 (26.5%)**	**Third-line therapy, *n* = 6672 (5.6%)**
**Aged ≥16 years**				
Nitrofurantoin	*n* (%)	84 407 (71.2)	11 741 (37.4)	1777 (26.6)
	Duration (days): mean (SD); median (IQR)	4.3 (2.9); 3 (3–5)	5.6 (6.1); 5 (3–7)	6.9 (9.0); 5 (3–7)
Trimethoprim	*n* (%)	17 187 (14.5)	6199 (19.7)	1093 (16.4)
	Duration (days): mean (SD); median (IQR)	4.8 (4.5); 3 (3–7)	6.2 (7.1); 5 (3–7)	8.7 (11.5); 7 (3–7)
Pivmecillinam hydrochloride	*n* (%)	3201 (2.7)	1924 (6.1)	459 (6.9)
	Duration (days): mean (SD); median (IQR)	21.9 (10.4); 28 (8–28)	21.7 (10.5); 28 (7–28)	21.3 (10.7); 28 (7–28)
Fosfomycin trometamol	*n* (%)	365 (0.3)	273 (0.9)	94 (1.4)
	Duration (days): mean (SD); median (IQR)	2.8 (6.4); 1 (1–1)	3.4 (7.2); 1 (1–2)	2.8 (6.0); 1 (1–2)
Amoxicillin	*n* (%)	4791 (4.0)	2916 (9.3)	653 (9.8)
	Duration (days): mean (SD); median (IQR)	6.5 (3.3); 7 (5–7)	6.6 (3.4); 7 (5–7)	6.8 (3.8); 7 (5–7)
		**First-line therapy, *n* = 1979 (100%)**	**Second-line therapy, *n* = 453 (22.9%)**	**Third-line therapy, *n* = 67 (3.4%)**
**Aged 12–15 years**				
Nitrofurantoin	*n* (%)	1157 (58.5)	168 (37.1)	23 (34.3)
	Duration (days): mean (SD); median (IQR)	3.8 (2.5); 3 (3–3)	4.8 (2.8); 3 (3–7)	4.5 (1.9); 3 (3–7)
Trimethoprim	*n* (%)	630 (31.8)	115 (25.4)	13 (19.4)
	Duration (days): mean (SD); median (IQR)	4.3 (3.3); 3 (3–5)	5.5 (4.7); 4 (3–7)	6.9 (6.6); 7 (3–7)
Amoxicillin	*n* (%)	69 (3.5)	40 (8.8)	10 (14.9)
	Duration (days): mean (SD); median (IQR)	6.9 (4.7); 7 (5–7)	7.4 (6.1); 6 (5–7)	10.4 (11.0); 5 (5–7)
Cefalexin	*n* (%)	41 (2.1)	35 (7.7)	9 (13.4)
	Duration (days): mean (SD); median (IQR)	7.4 (5.0); 7 (5–7)	10.3 (10.8); 7 (5–7)	8.0 (4.7); 7 (7–7)

IQR = interquartile range. SD = standard deviation.

Non-concordant treatment was less common in patients with re-prescription (35.0%) than without (44.1%), driven by discrepancies in therapy dose and/or duration. Patients requiring a re-prescription within 28 days of index were older, more likely to be menopausal, with a greater comorbidity burden, higher prevalence of recurrent uUTI at baseline, and greater exposure to antimicrobials during baseline. There were few region or practice-level differences between patients with or without re-prescription (see Supplementary Table S6).

The weighted regression model showed no significant increase in hospitalisation risk with non-concordant versus concordant treatment (OR 1.31, 95% confidence interval [CI] = 0.99 to 1.73, *P* = 0.06). Risk of hospitalisation was higher among patients who had an index visit at home, were older, and from economically deprived English regions (IMD quintiles 4 or 5) at index (see Supplementary Table S7).

### Objective 3: Economic burden

Overall, 103 544 patients with uUTI were age and comorbidity-matched 1:1 with 103 544 controls. Patient-, region-, and practice-level characteristics showed some imbalances between uUTI and control cohorts (see Supplementary Table S8).

**Figure 1.**
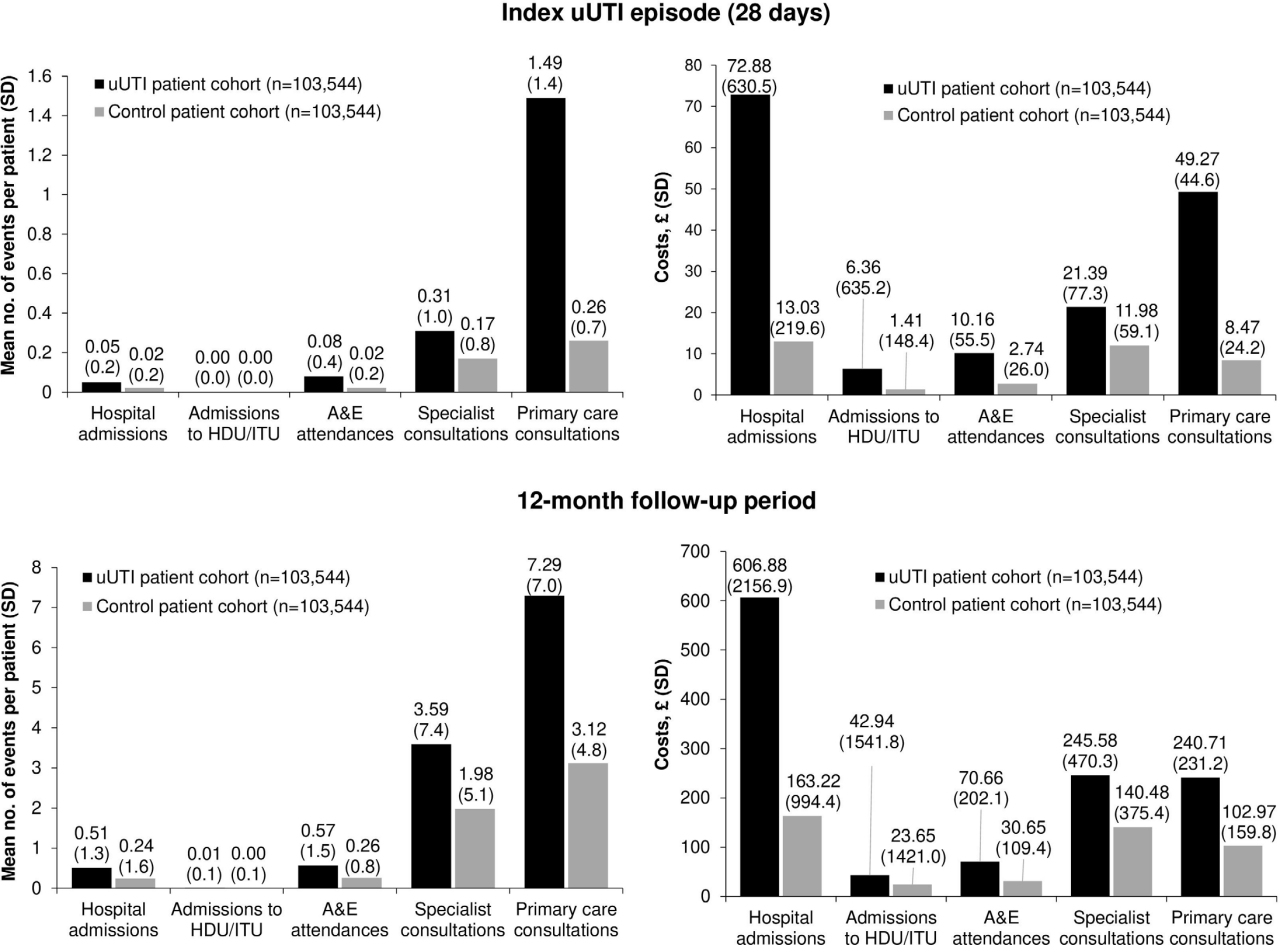
Mean all-cause HCRU and healthcare costs for the uUTI cohort and 1:1 matched control cohort, during the index uUTI episode and 12-month follow-up period. Mean (SD) number of events per patient. HCRU *P*<0.001, univariable negative binomial regression; this is owing to the over dispersion in the count data. Costs *P*<0.001, Mann–Whitney U test. *P*<0.001 applies to all comparisons between uUTI patients and controls for HCRU and costs. A&E = accident and emergency. HCRU = healthcare resource use. HDU = high dependency unit. ITU = intensive therapy unit. SD = standard deviation. uUTI = uncomplicated urinary tract infection.

Patients with uUTI had a higher HCRU risk in primary and secondary care versus controls (Figure 1). During the index episode and 12-month follow-up, patients with uUTI were at significantly higher risk than matched controls for all-cause hospital admissions, A&E attendances, specialist consultations, and primary care consultations (*P*<0.001, Table 4). During the 12-month follow-up, patients with uUTI were also at significantly higher risk than matched controls for HDU or ITU admissions (*P*<0.001).

**Table 4.** Determinants of hospitalisation and increased HCRU among controls compared with female patients with uUTI in England (Poisson model)

**Variable**	**During uUTI episode (28 days)**	
	**Controls versus patients with uUTI, risk ratio (95% CI)**	***P*-value**
All-cause hospital admissions	0.38 (0.36 to 0.40)	<0.001
All-cause HDU or ITU admissions	NA^a^	NA^a^
All-cause A&E attendances	0.31 (0.30 to 0.33)	<0.001
All-cause specialist consultations	0.56 (0.55 to 0.57)	<0.001
All-cause primary care consultations^b^	0.17 (0.17 to 0.18)	<0.001
**Variable**	**12-month follow-up**	
	**Controls versus patients with uUTI, risk ratio (95% CI)**	***P*-value**
All-cause hospital admissions	0.46 (0.45 to 0.47)	<0.001
All-cause HDU or ITU admissions	0.49 (0.42 to 0.56)	<0.001
All-cause A&E attendances	0.52 (0.51 to 0.52)	<0.001
All-cause specialist consultations	0.52^b^ (0.51 to 0.53)	<0.001
All-cause primary care consultations^b^	0.40^b^ (0.40 to 0.41)	<0.001

^a^All-cause admissions to HDU or ITU unit are not reported owing to convergence errors in modelling and so results were not reliable; this was likely owing to the small numbers of random variables. ^b^General practice. A&E = accident and emergency. HCRU = healthcare resource use. HDU = high dependency unit. ITU = intensive therapy unit. NA = not applicable. UTI = urinary tract infection. uUTI = uncomplicated urinary tract infection.

HCRU-associated costs were significantly lower among controls versus patients with uUTI, notably for primary care, specialist consultations, A&E visits, and hospital admissions (*P*<0.001, Figure 1). Note that distributions of HCRU and costs are overdispersed because controls have zero uUTI-specific costs in the 28-day follow-up. In both cohorts for both the index period and follow-up, visit costs were greatest for hospital admissions, but HCRU and associated costs were predominantly attributable to general practice consultations. uUTI impact was greatest on primary care provision: mean number of all-cause consultations at 28 days was 1.49 (SD 1.4) in uUTI patients versus 0.26 (SD 0.7) in matched controls; associated mean all-cause cost was £49.27 (SD £44.64) in uUTI patients versus £8.47 (SD £24.16) in controls. At 12 months post-index uUTI episode, mean number of all-cause primary care consultations was 7.29 (SD 7.0) in uUTI patients versus 3.12 (SD 4.8) in matched controls, with associated mean costs of £240.71 (SD £2313.17) versus £102.97 (SD £159.8). All-cause HCRU and costs were higher at both 28 days and in the 12-month follow-up in uUTI patients (data not shown). Mean all-cause costs for index episode and 28-day follow-up period were £160.06 for uUTI cases and £37.63 in controls. Mean all-cause cost over a 12-month follow-up period for the uUTI cohort was £1206.77 versus £460.97 in controls.

## Discussion

### Summary

uUTI progression to hospitalisation for acute pyelonephritis, bacteraemia, or sepsis was rare, with only 0.2% of patients requiring hospitalisation; this was driven by patient-level characteristics rather than region- or practice-level factors. These progression rates are lower than previous estimates, where <2% of women aged ≥65 years diagnosed with uUTI developed either urosepsis or acute pyelonephritis.^20^ Risk of hospitalisation was strongly associated with uUTI consultation at home (likely a surrogate for frailty or disease severity), as well as with increasing age, and hospitalisation or receipt of medication associated with comorbidities in the previous year. The proportion of patients requiring hospitalisation remained small, indicating patients were well-managed with medication. However, patients with uUTI had significantly increased short- and long-term primary and secondary HCRU and cost burden versus age and comorbidity-matched controls (*P*<0.001).

Based on available patient record data, over half of patients were prescribed a recommended antibiotic concordant with the latest English treatment guidelines.^8^ Non-concordance was driven by discrepancies in therapy dose and/or duration, and occurred in fewer patients with re-prescription versus those without. However, there was no significant increased risk of hospitalisation for patients receiving non-concordant versus concordant treatment (OR 1.31, 95% CI = 0.99 to 1.73, *P* = 0.06); although some patients received treatment not aligned with NICE lower UTI treatment guidelines, this was not associated with hospitalisation for uUTI disease progression (acute pyelonephritis, sepsis, or bacteraemia).

All-cause HCRU and associated costs were significantly higher in patients with uUTI versus matched controls (*P*<0.001), suggesting optimised patient management is needed to improve clinical outcomes.

### Strengths and limitations

Our results contribute significantly to current evidence characterising treatment patterns, disease progression, and HCRU in a large number of patients with uUTI in England, and are valuable given the paucity of data from outside the US. Being sourced from CPRD and HES,^13,21^ the data are broadly representative of, and generalisable to, real-world clinical practice in England. By linking the CPRD and HES, we could track the patient journey across primary and secondary care settings. However, this linkage is typically available among primary care practices focused on research, potentially limiting study generalisability.

Of note, this study may underestimate the number of patients with an index uUTI who progressed to acute pyelonephritis. Those patients who sought treatment (within the post-index window) for acute pyelonephritis in a primary care setting rather than the hospital or A&E setting were not captured in HES data. Other limitations include reliance on diagnosis coding and lack of clinical details from medical charts, microbiological laboratory results, and prescription data (HES does not contain pharmacy data). Thus, it was difficult to identify which patients were hospitalised directly or indirectly owing to uUTI caused by specific uropathogens. Furthermore, older patients with disease progression may have been coded with the immediate condition leading to hospitalisation (for example, fall) and not acute pyelonephritis, bacteraemia, or sepsis, therefore potentially underestimating uUTI progression. Information on clinical outcomes, such as treatment failure in patients hospitalised for uUTI progression, were not available in HES.

For the HCRU and cost analysis, patients were matched 1:1 on age and baseline CCI score category only, potentially resulting in residual confounding. Given the rarity of disease progression, the study may not have been powered to fully explore determinants for this outcome.

Concordance was ascertained using NICE guideline NG109 (published October 2018),^8^ but this study included uUTIs from January 2018 onwards. Although this could present a study limitation, previous recommendations under QS90 (urinary tract infections in adults [published June 2015])^22^ provided broad statements on antimicrobial prescribing overall, although a 3-day treatment regimen was recommended. A later update to QS90 also cross-referenced NG109 for NICE prescribing recommendations, indicating a level of agreement between the documents.

### Comparison with existing literature

Our study provides insight into the burden of community-acquired uUTI, where other studies focus on complicated UTI.^23^ Other studies report disease progression,^20^ optimal and suboptimal uUTI treatment,^24^ and associated disease burden findings in the US;^10,25,26^ we provide data for England. A recent smaller study examined risk factors for hospitalisation in East London patients with uUTI and observed increased odds of hospitalisation owing to UTIs with patient-level characteristics such as increasing age, prior antibiotics, and recurrent UTI.^27^ A recent NHS study highlighted the UK hospitalisation burden of UTI; between 2018 and 2023, 800 000 hospital admissions listed UTI as the primary diagnosis, rising to 1.8 million for admissions involving UTI.^28^ We note this dataset included patients with complicating comorbidities (pregnancy) and neonatal UTI, which were not included in our study (see Supplementary Table S1).

Compared with a previous study in the US, with similar inclusion and exclusion criteria, where 66.6% of patients were determined to have received treatment per uUTI Infectious Diseases Society of America guidelines,^29^ only 56.4% of patients in our study were classified as receiving treatment concordant with NICE lower UTI guidelines (NG109).^8^ In the US study, patients who received treatment not in accordance with the guidelines had greater all-cause and UTI-related costs versus those prescribed antibiotics concordantly.^29^

More than 70% of patients in our study received nitrofurantoin and nearly 15% trimethoprim, aligning with the first-choice antibiotics recommended by the NICE guidelines (NG109).^8^ These proportions are similar to those in the US study, where guidelines also recommend these antibiotics.^29^

### Implications for practice

The CPRD contained details of >120 000 patients in England diagnosed with uUTI between 2018 and 2019, with over two in five patients receiving non-concordant treatment. Potential consequences include re-prescription and recurrent uUTI, increasing the burden of illness. Our study showed the range of patient outcomes with uUTI, with a low percentage hospitalised owing to uUTI progression. This emphasises the importance of considering individual patient-level factors such as age, uUTI consultation at home, and potential comorbidities when treating uUTI to minimise disease progression risk. The overall burden of uUTI in terms of HCRU and costs, and existence of this substantial burden even though patients presenting with uUTI may appear unlikely to experience complications, highlights the need to optimise patient management.

## Data

This study is based in part on data from the Clinical Practice Research Datalink (CPRD) obtained under license from the UK Medicines and Healthcare products Regulatory Agency. The data are provided by patients and collected by the NHS as part of their care and support. The interpretation and conclusions contained in this study are those of the author/s alone. In addition, access to CPRD data is subject to protocol approval via CPRD’s Research Data Governance Process. CPRD is ultimately responsible for granting protocol approval, considering independent advice provided by Expert Review Committees and the Central Advisory Committee, as relevant. Therefore, these data cannot be made publicly available. The Hospital Episode Statistics database (copyright © 2021) was re-used with the permission of The Health & Social Care Information Centre. All rights reserved. The data supporting the findings of this study are available within the article.
